# All-you-can-eat: autophagy in neurodegeneration and neuroprotection

**DOI:** 10.1186/1750-1326-4-16

**Published:** 2009-04-06

**Authors:** Philipp A Jaeger, Tony Wyss-Coray

**Affiliations:** 1 Institut für Chemie und Biochemie, Freie Universität Berlin, Thielallee 63, Berlin, Germany; 2 Geriatric Research Education and Clinical Center, VA Palo Alto Health Care System, 3801 Miranda Ave, Palo Alto, California, USA; 3 Department of Neurology and Neurological Sciences, Stanford University School of Medicine, 300 Pasteur Ave, Stanford, California, USA

## Abstract

Autophagy is the major pathway involved in the degradation of proteins and organelles, cellular remodeling, and survival during nutrient starvation. Autophagosomal dysfunction has been implicated in an increasing number of diseases from cancer to bacterial and viral infections and more recently in neurodegeneration. While a decrease in autophagic activity appears to interfere with protein degradation and possibly organelle turnover, increased autophagy has been shown to facilitate the clearance of aggregation-prone proteins and promote neuronal survival in a number of disease models. On the other hand, too much autophagic activity can be detrimental as well and lead to cell death, suggesting the regulation of autophagy has an important role in cell fate decisions. An increasing number of model systems are now available to study the role of autophagy in the central nervous system and how it might be exploited to treat disease. We will review here the current knowledge of autophagy in the central nervous system and provide an overview of the various models that have been used to study acute and chronic neurodegeneration.

## Background

Cells have a constant need for the building blocks of life: amino acids, lipids, carbohydrates, and nucleic acids. To sustain this catabolic and anabolic need, they rely on uptake and recycling. While nutrient uptake is important, different degradation systems are in place to efficiently turnover recyclable intracellular material and provide quality control. The main pathways for protein degradation and recycling are the ubiquitin/proteasome pathway (for degrading short-lived cytosolic and nuclear proteins) [1], the lysosomal pathway (for cytosolic proteolysis), and autophagy (for bulk cytosolic degradation and organelle recycling) [2]. Deficits in any of these recycling pathways can result in uncontrolled accumulation of cellular debris or severe deficiencies in metabolic productivity, ultimately causing cell death.

The term autophagy, coined from the Greek words of αυτός ('autos', self) and φαγειν ('phagein'), meaning 'eating', was first used in 1963 by Christian de Duve to establish a nomenclature for different cellular pathways and compartments in the endosomal-lysosomal pathway [3]. Early work in autophagy research was done in rat liver cells and autophagy was characterized as a physiological response to starvation in order to degrade and recycle non-essential intracellular macromolecules [4-6]. Later, autophagy [7] and many of the autophagy genes [8] were identified in yeast, which gave the scientific community access to powerful cloning and pathway analysis tools. Subsequent identification of mammalian homologues led to the investigation of the role of autophagy in cancer, programmed cell death, tissue remodeling, heart, liver and muscle diseases, and bacterial and viral infections [9].

In recent years, increasing attention has been focused on the role of autophagy in metabolism of misfolded proteins and neuronal cell death in neurodegeneration (for comprehensive reviews see [10-13]). Abnormal autophagy has been implicated in the pathology of numerous diseases of the central nervous system (CNS), both chronic disorders (such as proteopathies) and many acute injuries. While it is still early in our understanding of this pathway, autophagy seems to have both beneficial and detrimental effects in disease, and it will be key to define the context that determines the outcome.

## Types of neuronal autophagy

Autophagy is involved in the intracellular turnover of proteins and cell organelles and has an important role in regulating cell fate in response to stress [14,15]. It is a highly conserved process that occurs in all species and cell types studied thus far. Two main types of mammalian autophagy have been identified and implicated in CNS injury and disease: macroautophagy and chaperone-mediated autophagy. Other more specialized forms of autophagy exist, such as mitophagy (direct targeting of mitochondria to lysosomes) [16], pexophagy (selective degradation of peroxisomes) [17,18], xenophagy (degradation of intracellular bacteria and viruses) [14,19], crinophagy (lysosomal fusion with re-directed exosomes) [20], microautophagy (direct engulfment of cytosol by lysosomes) [21,22], and piecemeal microautophagy of the nucleus (partial sequestration and degradation of the nucleus) [23], but most of them have only been observed in yeast or under special conditions and are not reviewed here.

**Figure 1:**
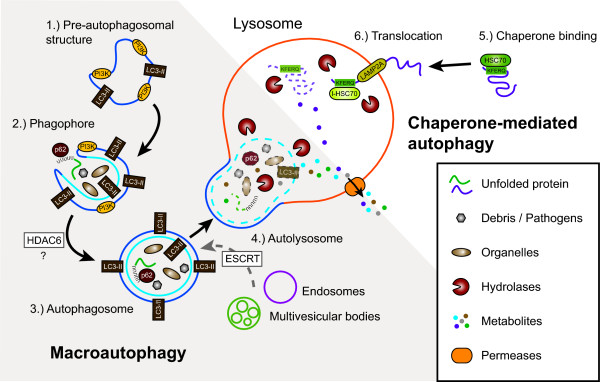
**Steps in macroautophagy and chaperone-mediated autophagy (CMA)**. Macroautophagy: 1.) Nucleation. An unidentified membrane source delivers lipid bi-layers for the formation of the phagophore. In yeast this early structure is termed pre-autophagosomal structure (PAS), its identity in mammalian cells is uncertain. A class III PI3K complex consisting of at least BECN1, PIK3C3, PIK3R4, UVRAG, and AMBRA1 is required for PAS formation and MAP1LC3 is anchored to the membrane via a phosphoethanolamine (PE) anchor (LC3-II). 2.) Expansion. The PAS or a comparable structure in mammals sequesters cytosolic cargo (either specifically via SQSTM1 [p62] or nonspecifically) by invagination, forming a double-membranous vesicle. This stage is also called "isolation membrane". More membrane and LC3-II is being recruited to the developing vacuole. 3.) Maturation. The completed autophagosome undergoes multiple maturation steps and fusion events with multi-vesicular bodies (MVB) or endosomes. The exact nature and sequence of this maturation, and whether these steps are always required is currently unknown. The autophagosomal lumen becomes more acidified during this maturation. 4.) Docking and fusion. During docking and fusion the inner membrane compartment together with its content gets released into the lysosome/autolysosome and is being degraded by lysosomal hydrolases. The components of the outer membrane are available for re-usage. Chaperone-mediated autophagy: 5.) Recognition and binding. The HSC70 chaperone complex (consisting of HSC70, HSP90 and maybe other proteins) recognizes unfolded proteins with the KFERQ sequence and moves them to the lysosome. 6.) Translocation. LAMP2A and a lysosomal form of HSC70 (l-HSC70) translocate the substrate protein across the lysosomal membrane into the lumen for degradation. The autophagy delivered substrates get degraded inside the lysosomes and their macromolecular components are made available to the cell's metabolism via permeases that allow their transport back into the cytosol.

**Macroautophagy **is a bulk degradation pathway and the only intracellular mechanism potentially capable of degrading large protein aggregates or damaged organelles. It is a well-understood process in yeast, but details about the exact sequence of events and the proteins involved are still uncertain in mammals. A cup-shaped isolation membrane forms around cytosolic components, eventually fusing to form a double membrane bound vesicle [24,25]. The origin of the membrane material for the formation of the isolation membrane is still under investigation, but recent evidence suggests that it might be derived from the endoplasmatic reticulum (ER) [26]. The protein MAP1LC3 is anchored via conjugated phosphatidylethanolamine (MAP1LC3-II) to the isolation membrane and is a specific marker for the so-called autophagosomes [27]. The autophagosome undergoes several microtubule- [28,29] and dynein-dependent maturation events [30,31], including fusions with multivesicular bodies (MVB), early and/or late endosomes [32,33], before it fuses with lysosomes [34,35] (Fig. 1, for a more comprehensive overview of autophagosome turnover see [28,36]).

**Figure 2:**
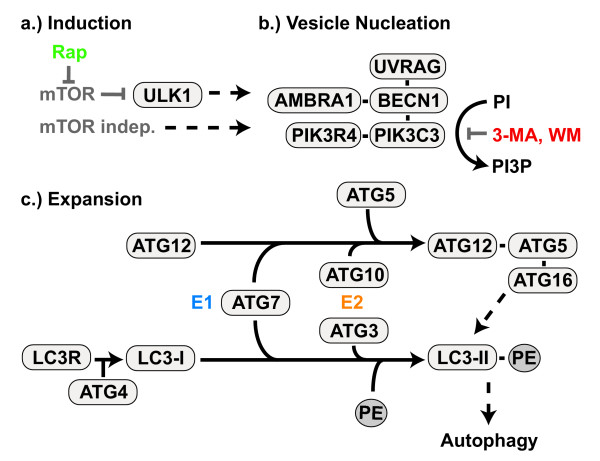
**Autophagy pathway in mammals**. The formation of autophagosomes appears to follow a pathway conserved across species and most findings made in yeast or other organisms also apply to mammalian autophagy. a.) Autophagy can be induced via mTOR dependent or independent pathways (for more information, see text and Fig. 3) which stimulate the nucleation and expansion of the phagophore/isolation membrane. b.) A multi-protein complex surrounding BECN1 with PI3K activity (mediated by PIK3C3) is important for the formation of the autophagosomal membrane. c.) Two ubiquitin-like modification systems are essential for mammalian autophagy; ATG12 is activated by ATG7 (E1 step), transferred to ATG10 (E2 step), conjugated to ATG5 and subsequently forms a complex with ATG16. This step is necessary early in autophagy for the formation of the phagophore or isolation membrane. MAP1LC3 (LC3) is cleaved by ATG4, activated by ATG7 (E1 step), transferred to ATG3 (E2 step), and conjugated to the phospholipid phosphoethanolamine (PE). This form known as MAP1LC3-II (LC3-II), localizes to the autophagosome membrane and is subsequently degraded in the lysosome. ATG4 cleaves off a C-terminal arginine (R) to expose a glycine residue that is then being linked to PE. Rapamycin (Rap) inhibits mTOR and activates macroautophagy, while 3-methyladenin (3-MA) and wortmannin (WM) inhibit the PI3K activity and de-activate macroautophagy.

At least 12 Atg (autophagy-related) and 4 other proteins are known to be involved in mammalian macroautophagy initiation and execution [37,38] (see Fig. 2). Whether direct autophagosomal-lysosomal fusion is possible, or endosomes first have to deliver essential enzymes to the maturating autophagosomes, is unclear. While the content of the autophagosome initially has the same pH as the surrounding cytosol, it becomes more acidic during its maturation [39,40]. For successful degradation of the autophagosomal content, autophagosomes need to migrate from their site of formation to lysosome rich peri-nuclear regions [29,41]. After fusion with the lysosome the outer autophagosome membrane can be reused, while lysosomal enzymes degrade the inner membrane and its cytosolic contents, enabling the recycling of macromolecules [42] (Fig. 1). It is unknown which markers, if any, label organelles or cytoplasm for sequestration and inclusion into autophagosomes. One possible marker for protein aggregates is the ubiquitin binding protein sequestosome 1 (SQSTM1, also known as p62) [43]. Almost all protein aggregates are poly-ubiquitinated and SQSTM1 binds both, MAP1LC3 and ubiquitin [44-46]. Macroautophagy components are expressed in neurons and neuronal cell lines (Tab. 1). While the function of autophagy-related proteins has been described for some, it is still unknown for others (Tab. 2). Macroautophagy has been implicated in chronic neurodegenerative diseases and acute neuronal injuries (Tab. 3 and 4).

**Table 1:** Presence of autophagy related gene expression in neuronal tissue.

**Gene**	**H. sapiens**		**M. musculus**		**R. norvegicus**	**Gene**	**D. melanogaster**	**Gene**	**C. elegans**
	mRNA	Protein		AllenB					
***ULK1***	[67]		[99,100,107]	Yes	[92]	***Atg1***	[97]	***unc-51***	WoBa [109,110,112,113]

***ATG3***	[74]		[104]	Yes		***Atg4/Aut1***	[111]	***atg-3***	

***ATG4***	[68]		[90]	Yes	[105]	***Aut2/Atg4***		***atg-4.1-2***	WoBa

***ATG5***			[60,91,93,96]	Weak	[87]	***Atg5***		***atg-5/atgr-5***	

***BECN1***	[59,69]	[59,65,69]	[59,81,96]	Yes	[80,94,95,106]	***Atg6***	[97]	***bec-1***	WoBa [116]

***PIK3C3***	[66]	[77]		Yes		***Vps34/Pi3K59F***		***vps-34/let-512***	[114]

***PIK3R4***	[71]			Weak		***Vps15/ird1***		***ZK930.1***	

***UVRAG***	[72]			Yes					

***AMBRA1***			[83]	n.a.					

***ATG7***	[75]		[61,96]	Weak	[75]	***Atg7***		***atg-7/atgr-7***	

***MAP1LC3***		[58,65]	[56,60,65,79,84,86,96]	Yes	[87-89,95,103,106]		[97]	***lgg-2***	

***GABARAP***	[78]			Yes	[89,92]	***Atg8a***		***lgg-1***	WoBa

***GABARAPL2***	[78]			Yes	[89]				

***ATG12***			[82,96]	Weak	[87]	***Atg12***		***lgg-3***	

***CHMP4B***			[85]	n.a.		***shrb/Vps32***	[108,115]	***vps-32.1***	WoBa

***HSPA8***	[70]	[63,73,76]		Yes	[76,98,101]	***Hsc70-4***		***hsp-1***	

***LAMP2***		[64]	[102]	Weak	[102,103]				

Examples of autophagy related gene expression in humans and common model organisms (mRNA and/or protein). For human, mouse, and rat genes the approved human gene symbol is used , for D. melanogaster and C. elegans their respective gene symbols (if existent) are provided. (*AllenB): *Gene mRNA is detectable by hybridization as published in the Allen Brain Atlas ; (*WoBa): *neuronal expression data available at WormBase ; *(n.a.): *not available.

**Table 2:** Neuronal phenotype of autophagy related knockout/knockdown animal models.

**Gene** **(Alias)**	**Protein function**	**Knockout/knockdown**	**OE/TG**	**ES/M @ IMSR**	**Neuronal phenotype after k.o./k.d**. **(Animal model)**	**K.o. embryonic lethal**
***ULK1*** ***(ATG1)***	Ser/Thr protein kinase (regulation and vesicle formation)	[107,112,113] * [97,99,100,131,132,135,141,145]	[140] (OE)	ES M (GT)	Impaired endocytosis of nerve growth factor, excessive axon arborization, stunted axon elongation (MM) Paralysis, aberrant axon growth, abnormal vesicles, arrested differentiation (CE)	Yes (DM)

***ATG3***	Ubiquitin-conjugating-like enzyme (attaches MAP1LC3 to PE)	[111,143]		n.a.	Not reported	Yes (DM)

***ATG4***	Cystein protease (cleaves C-terminus of MAP1LC3 for conjugation)	[90,144]		ES M (GT/TG)	Not reported	Yes (CE)

***ATG5***	Unknown (conjugates to ATG12, binds ATG16)	[60] * [91,130,141]		ES M (MUT)	Progressive motor deficits, accumulation of inclusion bodies, neurodegeneration, aberrant vacuoles in Purkinje cells (MM)	No # (DM/MM)

***BECN1*** ***(ATG6)***	Unknown (part of class III PI3K complex, anchor protein, autophagy initiation)	[59] * [97,116,124,137,146]	[119] (TG)	M (TG)	Neurodegeneration, lysosomal abnormalities (MM)	Yes (MM/CE/DM)

***PIK3C3*** ***(VPS34)***	Class III PI3K complex (forms complex with BECN1/PIK3R4/AMBRA1/UVRAG, autophagy initiation)	[123,142] * [114,126]		ES	Abnormal protein aggregation, abnormal locomotion (CE)	Yes (CE)

***PIK3R4*** ***(VPS15, P150)***	Ser/Thr protein kinase (forms a complex with and activates PIK3C3)	[134]		ES	Not reported	Yes (DM)

***AMBRA1***	Unknown (component of the class III PI3K complex)	[83] *		ES	Neural tube defects, polyU aggregates, unbalanced cell proliferation, cell death (MM)	Yes (MM)

***ATG7***	Ubiquitin-activating-like enzyme (activates MAP1LC3 and ATG12 for conjugation)	[61,123,129] * [124,125,127,128,136,141]		ES	Behavioral deficits, neuronal loss, polyU inclusions, axonal dystrophy, axonal terminal degeneration (MM) PolyU aggregates, neuronal degeneration (DM) Abnormal protein aggregation (CE)	No # (DM/MM)

***MAP1LC3*** ***(LC3)***	Unknown (similarity with ubiquitin, part of autophagosomal membrane)	[123,145] * [79,97]	[27] (TG)	ES M (TG)	Abnormal protein aggregation (CE)	Yes (CE) No (MM)

***ATG12***	Unknown (similarity with ubiquitin, conjugated to ATG5)	[123] *		n.a.	Abnormal protein aggregation (CE)	Unknown

***CHMP4B*** ***(SNF7-2)***	Unknown (part of the ESCRT-III complex, involved in surface receptor degradation, formation of MVBs and autophagosomes)	[85,115,138]		ES	Dendritic and axonal branching impaired, dendritic retraction, reduced cell viability, autophagosomes accumulate, increased htt toxicity (DM)	Yes (MM)

***HSPA8*** ***(HSC70)***	Chaperone (recognizes CMA motif, lysosomal translocation)	[121,139] *	[120] (OE)	ES	Impaired transmitter release, o.e. rescues α-synuclein pathology, Bolwig's nerve projection abnormalities (DM)	Yes (DM)

***LAMP2***	Unknown (Lysosomal membrane glyco-protein, forms complex with HSPA8)	[40]		ES	Not reported	No (MM)

Examples of model organism with knockout, knockdown, or overexpression of autophagy genes and the corresponding neuronal phenotype. Approved human gene names are used , in addition commonly used aliases are provided. # While non-neuronal *Atg5 *and *Atg7 *k.o. mice survive birth, they die within one day postnatal. *(MM): *M. musculus; *(DM): *D. melanogaster; *(CE): *C. elegans; *(OE): *overexpression; *(ES): *embryonic knockout stem cell; *(M): *mouse line; *(TG): *transgenic; *(GT): *gene-trap; *(MUT): *targeted mutation; *(IMSR): *knockout ES/mice available through the International Mouse Strain Resource ; (*): neuronal tissue examined; *(n.a.): *not available.

**Table 3:** Autophagy in common chronic neurodegenerative diseases.

**Disease**	**Autophagosomal phenotype**	**Ref**.
**Alzheimer disease**	Autophagy appears impaired, autophagosomes accumulate, endosomal-lysosomal abnormalities, increased mitophagy, reduction of macroautophagy enhances pathology, pharmacological activation of macroautophagy can promote the clearance of Aβ/APP and reduces tau pathology, autophagosomes contain APP/Aβ/secretases.	[206,208,59,62,204,207,203-209-205,57,58,118]

**Parkinson disease**	Autophagy/mitophagy appears impaired, autophagosome-like structures accumulate, pharmacological activation of macroautophagy enhances α-synuclein clearance and is neuroprotective, α-synuclein is a target of CMA and macroautophagy and the proteasome, dopamine-modified/mutated α-synuclein blocks CMA and dopamine induces autophagic cell death and α-synuclein accumulation, mutant UCH-L1 binds to LAMP2A and inhibits CMA.	[220,214,213,219,212,102,192,210,211,218,217,117]

**Huntington diseases**	Impaired sorting/degradation of autophagosomes, autophagosomes accumulate, BECN1 is recruited to htt inclusions and BECN1 reduction causes enhanced htt accumulation, pharmacological or signaling mediated activation of macroautophagy reduces htt toxicity, mTOR is sequestered into htt inclusions, which causes macroautophagy activation.	[225,227,216-231,203,221,226,224,195,223,222]

**Frontotemporal dementia**	Impaired endosome maturation, enlarged autophagosome accumulation, mutant CHMP2B disturbs the ESCRT-III complex for endosomal sorting which results in polyU/SQSTM1 aggregates.	[162,85]

**Amyotrophic lateral sclerosis**	Impaired early endosomes, impaired sorting/degradation of autophagosomes, CHMP2B disturbs the ESCRT-III complex for endosomal/MVB sorting which results in polyU/SQSTM1 aggregates, MVBs are required for TDP-43 clearance, Lithium activates protective autophagy.	[232,86,162,233]

**Table 4:** Autophagy in acute neuronal injury.

**Injury**	**Autophagy related changes**	**Ref**.
**Hypoxia/Ischemia**	Mixed results after hypoxic treatments: Knockout of Atg genes in C. elegans decreases survival after hypoxia and autophagy activation by rapamycin treatment leads to injury reduction in rat and rat tissue. On the contrary, *Atg7*^-/- ^mice lacking functional autophagy in the CNS are largely protected from neurodegeneration.	[247,80,104,94,244,246,245]

**Trauma**	Macroautophagy appears to be beneficial: Autophagy can be activated for more than a month following brain trauma (elevated BECN1, MAP1LC3-II, ATG5-12 levels, increased AV numbers) in rodents, autophagy appears activated in human tissue samples. Rapamycin treatment is neuroprotective in mice.	[106,87,249,248,65,95,84,81,250]

**Pharmacological injury**	Autophagy appears to be deleterious: Transient activation of autophagy after injury (elevated MAP1LC3-II, p-mTOR, LAMP2, increased AV numbers) and activation of apoptosis in rodents and primary neuronal culture. 3-MA treatment or RNAi against *ATG5 *or *BECN1 *blocks cell death.	[96,252,166,103,251,254]

**Trophic deprivation**	Autophagy appears to be deleterious: Growth factor withdrawal leads to autophagic cell death in rodents or chicken, 3-MA blocks cytochrome C release and delays apoptosis.	[257,255,256,259-258]

**Chaperone-mediated autophagy **(CMA) is distinctly different from macroautophagy in that no vesicular trafficking is involved (Fig. 1). Instead, a pentapeptide motif in substrate proteins allows their specific translocation to the lysosome membrane (reviewed in [47-49]). Thus, CMA degrades only proteins with the motif KFERQ or a biochemically related sequence, which is present in about 30% of all cytosolic proteins [50]. It has recently been suggested that 80% of aminoacyl-tRNA synthases are also substrates for CMA [48], indicating a possible role of CMA in protein synthesis control under starvation conditions.

To be targeted for CMA, substrate proteins first bind to a cytosolic complex containing the chaperone HSC70 (Fig. 1). This complex then interacts with a lysosomal membrane complex containing LAMP2A and HSP90 [51]. The substrate protein is finally degraded after unfolding and translocation into the lysosomal lumen (with the help of lys-HSC70, a luminal form of HSC70) [51]. The chaperone complex consists of many more proteins but their exact localization and role in CMA is presently unclear [52].

Macroautophagy and CMA are interconnected, although the details of this crosstalk are not well understood. A possible connection is BCL2 associated athanogene (BAG1) which functions as a nucleotide exchange factor for HSC70 [53] and has been reported to bind MAP1LC3 [54]. Impairing macroautophagy, either genetically or pharmacologically, results in a compensatory up-regulation of CMA [55]. CMA components are expressed in neurons and neuronal cell lines (Tab. 1) and CMA has also been implicated in chronic neurodegenerative diseases (Tab. 3).

## Autophagy in the healthy nervous system

The brain is well protected against short-term periods of systemic starvation. Selective transport of glucose, amino acids, and hormones across the blood-brain-barrier ensures ample supply of metabolites and local populations of glia cells release trophic factors under normal or energy restricted conditions. High levels of constitutive autophagy in neurons may therefore not be necessary to maintain the cellular energy needs; indeed, forty-eight hours of food deprivation caused no apparent autophagy induction in the mouse brain [56].

Instead, autophagy probably supports local housekeeping functions within the neuron: macroautophagy is the only cellular mechanism capable of degrading expired organelles in neurons that can live for decades. In addition autophagy is a potential clearing mechanism for protein aggregates that occur frequently in aging neurons, but not in young and healthy cells. Consistent with such a role in the normal brain autophagosome numbers [57] and the levels of MAP1LC3-II protein [56,58,59] are low when compared with other tissues. Nevertheless, recent findings show that autophagy in neurons is indeed constitutively active [60,61] and autophagosomes accumulate rapidly when their clearance is blocked [62], indicating fast basal turnover.

A number of autophagy related genes are expressed (measured either by mRNA or protein analysis) in neuronal tissues of humans [58,59,63-78], rodents [56,59-61,65,75,76,79-107], and insects [97,108-116] (Tab. 1). Electron microscopy of human and mouse brain tissue shows the presence of lysosomes and autophagosomes in neurons further supporting a basal level of autophagy during normal neuronal homeostasis [57,58,117,118]. Model organisms have been crucial for the identification of genes that regulate autophagy and clarification of their function as detailed in Tab. 2[27,40,59-61,79,83,85,90,91,97,99,100,107,111-116,119-146].

Age is a major risk factor for many neurodegenerative diseases and a number of studies suggest a role for autophagy in aging. Interestingly, protein degradation and specifically autophagy (both macroautophagy and CMA) decline with age, although to what extent that reduction occurs within the CNS is not clear [147-150]. An age related decline of Atg genes has been shown in *D. melanogaster*, and Atg8 overexpression increases the fly's lifespan [151,152] while RNAi of autophagy genes in *C. elegans *leads to decreased lifespan [136,153]. If and how decreasing autophagy activity in the aging human CNS contributes to the higher prevalence of neurodegenerative diseases and accumulation of various protein aggregates will have to be clarified in future studies.

## Autophagy as a clearing mechanism for protein degradation

The strongest evidence for an active role of autophagy in maintaining neuronal homeostasis comes from engineered mutant mice lacking autophagy genes. While *Atg5 *and *Atg7 *knockout mice had been created before [128,130], their early developmental mortality made the study of the adult CNS impossible. To overcome this limitation, two landmark studies generated conditional knockout mice lacking *Atg5 *and *Atg7 *only in neurons [60,61].

The *Atg5*^flox/flox^;nestin-Cre mice showed growth retardation, progressive motor and behavioral deficits, prominent neurodegeneration and axonal swelling in a number of brain regions. Histological examination also revealed abundant ubiquitin-positive inclusions in neurons, indicating a crucial role of autophagy in the turnover of diffuse cytosolic proteins labeled for degradation [60].

In the *Atg7*^flox/flox^;nestin-Cre mice, strikingly similar pathological changes occurred: reduced growth, motor and behavior changes, loss of Purkinje cells, activation of glia cells, and accumulation of ubiquitinated inclusions. Proteasomal function was not impaired by autophagy inhibition, which shows that autophagy has an important role in the basal turnover of poly-ubiquitinated (polyU) proteins together with the proteasome [61]. The ubiquitin-positive aggregates also contain abnormal amounts of SQSTM1 [127].

While polyU proteins themselves are sticky but not highly aggregating, the presence of large amounts of SQSTM1 might enhance their aggregation [43,154]. SQSTM1 can directly interact with MAP1LC3 [45] and tags ubiquitinated protein-aggregates for autophagic degradation [43,155]. It appears that impairment of autophagy leads to the accumulation of SQSTM1, which in turn increases the rate of aggregation for diffuse ubiquitinated proteins. Interestingly, the double knockout of *Atg7 *and *Sqstm1 *prevents the formation of ubiquitinated aggregates in neurons, but has no effect on the other observed neurodegenerative phenotypes [127], indicating that autophagy plays multiple roles in neuronal homeostasis, not just clearance. This crosstalk between autophagy and the ubiquitin-proteasome system (UPS) is supported by *in vitro *induction of autophagy in response to impaired UPS [156]. SQSTM1 is not the only protein facilitating the degradation of protein aggregates via autophagy, as HDAC6, a microtubule-associated histone deacetylase that interacts with polyU proteins, also provides a link to autophagy (see below [156,157]).

Additional evidence for a role of autophagy in protein turnover comes from mice lacking *Ambra1*, a recently discovered regulator of autophagy that interacts with Beclin 1 (BECN1) [83] (Fig. 2). *Ambra1 *knockout mice show polyU inclusions and severe neural tube deficits, unbalanced cell proliferation, and excessive apoptotic cell death. Autophagy has a complex interplay with apoptosis, where it can serve both as an alternative cell-death and as an anti-apoptotic survival mechanism. More details of this relationship will be discussed at the end of this article and comprehensive reviews have been published on this topic elsewhere [133,158].

## Autophagy in vesicle sorting and organelle turnover

Another set of important findings indicates that endosomal sorting and endosomal-autophagosomal fusion are impaired in certain neurodegenerative diseases. ESCRT-0 to III (endosomal sorting complex required for transport) orchestrate the progression of endosomes along the endosomal-lysosomal pathway. Dysfunction of one of these complexes (ESCRT-III), either by RNAi depletion of its essential subunit *CHMP4B *(also known as *SNF7-2*) or by expression of a mutant CHMP2B protein (another subunit of ESCRT-III and associated with Frontotemporal dementia linked to chromosome 3), caused autophagosome and polyU protein aggregate accumulation, and dendritic retraction followed by neuronal death in cultured mature cortical neurons [85]. It has been suggested that the endosomal and autophagosomal pathways merge upstream of lysosomal fusion [159-161], in particular that intact multivesicular bodies (MVB) are essential for autophagosome maturation [138,162]. ESCRT-III seems to play an important role during this endosomal-autophagosomal fusion event and its dysfunction leads to impaired processing and accumulation of autophagosomes. In a recent paper, deletion of the *Hrs *(also known as *Hgs*) gene, a component of ESCRT-0, in the neurons of *Hrs*^flox/flox^;SynI-cre mice caused apoptosis, loss of hippocampal CA3 pyramidal neurons, and accumulation of polyU proteins and SQSTM1 [163]. Accordingly, locomotor activity and learning ability were severely reduced in these mice.

While no evidence for the autophagosomal degradation of specific neuronal organelles (such as synaptic vesicles) in healthy neurons exists thus far, mitochondria were selectively degraded by macroautophagy in neurons exposed to experimental neurotoxins 1-methyl-4-phenylpyridinium (MPP+) or 6-Hydroxydopamine, which induce mitochondrial damage [164,165]. Autophagosomes were also observed in dopaminergic neurons treated with methamphetamine [166], supporting the idea that autophagy serves to clear damaged organelles in neurons. Together, these studies underline the critical role of autophagosomal-endosomal-lysosomal trafficking and sorting in neuronal homeostasis

## Autophagosomes as transport vacuoles

Autophagosomes are not only found in the soma but also in the distal parts of the axon and dendrites and can be retrogradely transported to the cell soma for degradation [167]. Autophagy may thus support neurite and growth cone remodeling and clear axons and dendrites of defective larger structures. Efficient bi-directional transport along the axon is necessary for neuronal survival [168,169] and supports the clearing of protein aggregates by autophagosomes [31].

In addition, autophagosomes are retrogradely transported, making them potential transport vacuoles for the delivery of trophic factors from the synapse to the cell body. Autophagosomes can travel along microtubules, possibly facilitated through an interaction between MAP1LC3 and MAP1A/B [29,41]. Some evidence exists that signaling endosomes containing nerve growth factor (NGF) might be derived from or be related to autophagosomes, based on the microscopic association of fluorescently labeled LC3 with retrogradely transported NGF and the NGF receptors TrkA and p75 [170]. This finding could indicate that disturbed autophagy (for example, as a result of changes in APP expression or metabolism) might contribute to the reported impairment of NGF transport in neurodegenerative diseases such as Down's syndrome. In this condition, an extra copy of chromosome 21, which contains the *APP *gene, results in increased APP expression and the development of Alzheimer-like dementia. Intriguingly, in a trisomic mouse model of Down's syndrome deletion of one copy of *APP *led to a marked improvement in transport of signaling endosomes containing NGF, reduced neurodegeneration, and improved cognitive function [171].

Several studies point towards an important role of ULK1 in this trafficking role of autophagy. For example, knockdown of *ULK1 *by RNAi in cultured mouse spinal sensory neurons leads to impaired endocytosis of NGF [107]. Axonal growth appears stunted in *C. elegans *in *unc-51 *mutants [110,112] and after *ULK1 *knockdown in mouse neuronal cells [107], while dominant negative *ULK1 *mutants expressed in immature murine cerebellar granule cells lead to inhibition of neurite outgrowth and developmental arrest [99]. ULK1 is important for autophagy initiation and has been reported to interact with GABARAP and GABARAPL2 (also known as GATE16), two homologues of MAP1LC3, in mouse pyramidal, mitral, and Purkinje cells. This interaction indicates an involvement of autophagosome transport in some of the *ULK1 *knockdown phenotypes [92], although it clearly has functions independent of autophagy [100,172].

Another interaction between autophagy and neuronal receptors was found in Lurcher mice, which have a mutation in the glutamate receptor GluRδ2 and are a model for ataxia. The mutated receptor GluRδ2^Lc^, but not the wildtype receptor, bind to BECN1 and may thus trigger autophagy in dying Purkinje cells in Lurcher mice [173,174]. In this way, autophagy might serve as an early stress response to axonal dystrophy. Autophagosomes appear rapidly in axons in Lurcher mice and this is attributed to the induction and local synthesis of autophagosomes in axon terminals in response to stress [174]. How autophagosomes form so fast in distal cell parts is unclear, but early ultrastructural studies suggest that smooth ER in axons might be a source for quick membrane supply [175,176].

## Regulation of autophagy

**Figure 3:**
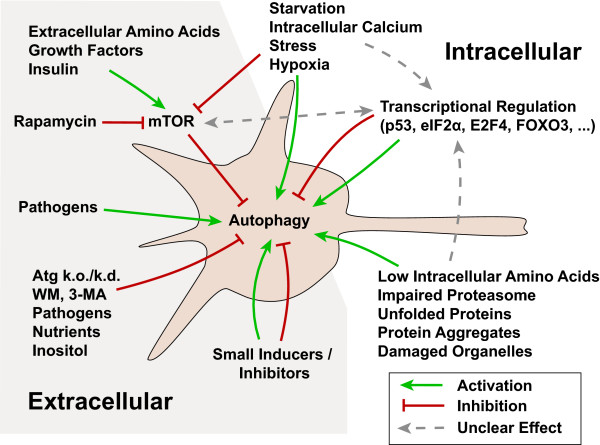
**Control of autophagy**. Autophagy is a major housekeeping pathway and under the control of many different signaling cascades. Mammalian Target of rapamycin (mTOR) plays a central role in the regulation of autophagic activity as it integrates signaling from different sensors of cellular homeostasis. When mTOR is active in yeast it keeps an important ULK1 binding partner (ATG13) phosphorylated, thus inhibiting the induction of autophagy. While signals indicating abundant nutritional and trophic support activate mTOR (and deactivate autophagy), signals of starvation or other stressors inhibit mTOR (and activate autophagy). Autophagy can be directly stimulated by intracellular debris (such as unfolded proteins and damaged organelles) or by indicators of an overwhelmed ubiquitin-proteasome system (UPS). Also certain pathogens activate autophagy. Autophagy can be directly inhibited by genetic ablation of important Atg genes, inhibitors of the class III PI3K-complex (WM, 3-MA), high nutrient levels, and inositol signaling. More recently screenings of small compound libraries have yielded inducers and inhibitors of autophagy, both mTOR dependent and independent. And last, transcriptional regulators, such as p53, eIF2α, E2F4, or FOXO3 regulate autophagy by controlling the expression levels of many Atg genes. For further details, please refer to the text.

Because of its key function in cell homeostasis, multiple signaling cascades have been implicated in the regulation of autophagy (Fig. 3). A large amount of this knowledge has been acquired in yeast and it is unknown how much can be translated to mammalian cells (for reviews see [177-179]). One of the key regulators of autophagy is the level of amino acids, both extracellular and intracellular. Cells measure intracellular amino acid levels via the protein kinase EIF2AK4 (also known as GCN2), which is activated by unloaded transfer RNAs. Low levels of intracellular amino acids leading to free transfer RNAs thus activate autophagy through phosphorylation of the eukaryotic initiation factor eIFα2 [180]. Extracellular amino acids are sensed via a putative receptor in the cell membrane [181], which seems to signal through mammalian target of rapamycin (mTOR, also known as FRAP1). mTOR is a protein kinase that plays a central role in nutrient sensing, cell proliferation, and metabolism [182-184], integrating many signaling pathways. Activated mTOR promotes protein synthesis and inhibits autophagy via phosphorylation of the ULK1 binding partner ATG13, while deactivated mTOR activates autophagy [185]. Insulin and growth factors signal through AKT, activate mTOR [182,186] and deactivate autophagy, while energy depletion [187] or elevated intracellular calcium [188] inhibit mTOR through AMP-activated protein kinase (AMPK) and activate autophagy. Other signaling cascades implicated in the regulation of autophagy include Ras/Raf and ERK signaling (mTOR dependent [189] or independent [190]) and the mTOR independent inositol signaling pathway [191,192]. Lastly, autophagy may be induced "directly" through the presence of intracellular inclusions [193-195]. It is unclear which of these pathways are involved in neurodegenerative conditions.

Even less is known about the transcriptional control of autophagy, especially in neurons. Nevertheless, a number of important transcription factors have been associated with the regulation of autophagy genes in non-neural cell types. Since these processes are likely conserved, they may contribute to the control of autophagy in neurons as well.

In one study, a high-affinity E2F4 transcription factor-binding region in the *BECN1 *promoter was identified [196]. A number of autophagy proteins are also controlled by the FOXO3 transcription factor in muscle cells [197,198] and potentially hepatoma and pheochromocytoma cells [198]. In these cell types, FOXO3 binds directly to the promoters of *MAP1LC3*, *ATG12*, and *GABARAP *genes to increase their expression and induce autophagy [197]. Indeed, FOXO3 increases the expression of ATG4, PIK3C3 and BECN1, but the exact mechanisms are unknown [198].

Members of the p53 family also play important roles in autophagy control: Cytosolic p53 inhibits autophagy [199], whereas nuclear p53 activates it [200]. The localization of p53 appears to be a sensor for genotoxic stress. In addition, p53 acts upstream of mTOR, inhibiting its activity through AMPK, thus stimulating autophagy. Recently, a p53 homologue, p73, has been identified by integrating whole-genome chromatin immunoprecipitation and expression profiling in cell culture that binds to regulatory regions of several autophagy genes (*ATG5, ATG7, UVRAG, GABARAP, AMBRA1, ATG16, PIK3C3*) presumably through its nuclear activity [201,202]. Further studies that investigate the upstream control of autophagy in neurons will greatly help to improve our understanding of the potential misregulation of autophagy during neurodegeneration.

The above findings suggest three main roles for autophagy in neuronal homeostasis: First, impaired autophagy results in abnormal protein aggregation across species, indicating an involvement of autophagy in the clearance of intracellular protein aggregates, especially when these aggregates are poly-ubiquitinated. Second, changes in vesicular appearance and trafficking point towards a crucial role of autophagy in maintaining the normal turnover and flux of vacuolar compartments and possibly trophic factors through the neuron. And third, disrupted autophagy leads to changes in neuronal morphology and connectivity, such as excessive axon arborization, stunted axon growth, axonal dystrophy, axonal terminal degeneration or impaired axonal projections, implicating autophagy genes and their gene products in neuronal shaping, connectivity, and development. Whether these observations are always directly linked to the gene's role in autophagy or are sometimes a result of non-autophagic functions remains to be determined.

## Autophagy in CNS disease and injury

Several excellent reviews have recently covered the emerging relationship between autophagy and various neurodegenerative diseases [10-13] and we provide only a brief overview of the most prevalent diseases associated with histopathological changes in autophagy. Instead, we summarize here which aspects of autophagosomal pathology that have been observed in human disease are now being successfully replicated in model systems (Tab. 3 and 4).

In general, the effect of autophagy in neurons during disease can be broadly divided into two classes: autophagosomal degradation is either impaired or excessively activated, leading to an apparent disruption of the intracellular organelle organization and accumulation of autophagosomes in neurons over long periods of time (chronic conditions, Tab. 3), or autophagy genes are activated in response to temporary injury/stress (acute response, Tab. 4).

## Autophagy in chronic CNS diseases

Typical examples of the first class of diseases are Alzheimer (AD) [57-59,62,118,203-209], Parkinson (PD) [102,117,192,210-220], and Huntington disease (HD) [195,203,216,221-231] (Tab. 3). In these diseases, the pathological accumulation of autophagosomes/autophagosome-like structures and abnormalities in the endosomal-lysosomal pathway were documented by electron microscopy (EM) in human postmortem brain tissue [57,58,117,118,207]. Diseases with a seemingly more endosomal pathology, but an autophagic component, are Amyotrophic lateral sclerosis (ALS) and Frontotemporal dementia (FTD) [85,86,162,232,233].

In Alzheimer research, expression analysis revealed that *BECN1 *mRNA is reduced in AD brain tissue [59,234], and BECN1 protein levels are significantly lower in the cortex of AD patients compared with age-matched controls [59]. This is despite the fact that an increase in autophagosome numbers in neurons from AD patients is obvious by EM, and AD brains also show increased levels of MAP1LC3-I and MAP1LC3-II [58]. A possible explanation for this apparent contradiction is that reduced BECN1 levels lead to changes in autophagosomal flux. This in turn could impair endosomal-lysosomal degradation, leading to a built-up of intracellular vesicular compartments over time. Changes in the endosomal-lysosomal pathway are amongst the earliest changes in AD [235] and a possible indicator for disturbed vacuolar trafficking.

While the aforementioned studies were descriptive, one of the first mechanistic insights into the possible role of autophagy in neurodegenerative diseases was provided by a study of primary neurons from a mouse model for HD. The authors observed increased autophagy, increased oxidative stress, and polyU aggregates in cultured striatal neurons from transgenic mice expressing mutant human huntingtin in response to a single exposure of a neurotoxic concentration of dopamine [223]. The results suggest that dopamine triggered free radical-mediated oxidation of macromolecules and stimulated autophagy. Subsequent studies demonstrated that SQSTM1 extensively decorates polyU protein aggregates, co-localizes with MAP1LC3 and becomes sequestered in autophagosomes. This highlights the importance of autophagy as a degradative pathway for polyU aggregates [43]. Another link between autophagy and protein aggregates was provided by a study showing that mTOR accumulates in huntingtin aggregates in cells, mice, and human brains [226]. The authors speculate that mTOR can be sequestered and inactivated in this way, leading to a protective induction of autophagic degradation of protein aggregates. Arguing against this interpretation is the observation that BECN1, a protein necessary for the induction of autophagy, is recruited into pathological huntingtin aggregates in human brain tissue as well [230].

The effect of autophagy on the degradation of protein aggregates was investigated further in cell culture and animal models using pharmacological inducers and inhibitors of autophagy (see Tab. 4). It was discovered that rapamycin, an inducer of autophagy, leads to the clearance of polyQ/polyA aggregates in cell culture, fly, and mouse models of HD [195,226]. This finding was confirmed for α-synuclein in cell culture [218] and wildtype tau in flies [203]. Together, these results triggered a concerted research effort to find mTOR dependent and independent pharmacological inducers of autophagy and led to the discovery of many small compounds that facilitate the clearing of aggregated proteins [216,219,229,236]. While pharmacological autophagy stimulation reduces the toxicity of many aggregate-prone proteins, experiments in cell culture demonstrate that α-synuclein can be degraded by both the proteasome and autophagy. Pharmacological inhibition of either pathway leads to increased intracellular α-synuclein levels [218]. Interestingly, pharmacological inhibition of microtubule formation by nocodazole treatment inhibits polyQ aggregate formation and at the same time increases its toxicity in cell culture [237,238]. This is at least partially due to the inhibition of autophagosome-lysosome fusion [239], demonstrating that intracellular transport is essential for proper aggresome/inclusion body formation and autophagosomal function. Furthermore, activation of autophagy through starvation in primary cortical mouse neurons expressing polyQ proteins protects against cell death [186]. In summary, autophagy might be especially effective in clearing aggregated proteins.

While these pharmacological studies increase our understanding of some aspects of autophagy in neurodegeneration, they mostly employ drugs that are rather nonspecific and they target proteins such as mTOR and AKT, which have broad functions outside autophagy. Genetic or RNAi-based methods overcome some of these limitations.

It has been shown, for example, that cytosolic protein aggregates can be specifically targeted by autophagy and that their aggregation increases after inhibition of autophagy by siRNA knockdown of *MAP1LC3 *in cell culture [221]. In *C. elegans*, RNAi mediated deletion of *bec-1*, *atgr-7*, and *Ce-atg18 *led to increased accumulation of polyQ aggregates in models for HD, confirming the earlier studies in mammalian cell culture systems [124].

The cytoplasmic histone deacetylase HDAC6, although not directly an autophagy related protein, plays an essential role in the microtubule- and dynein-dependent intracellular movement of polyU protein aggregates [240]. *HDAC6 *RNAi impairs retrograde transport of autophagosomes and lysosomes [156]. HDAC6 overexpression, on the other hand, is sufficient to rescue neurodegeneration caused by proteasome mutations or polyQ toxicity in transgenic flies via autophagy, providing a direct link between UPS and autophagy [157]. HDAC6 activates autophagy by an unknown mechanism, leading to accelerated protein turnover. Potential mechanisms include modulation of HSP90 (and maybe CMA), a substrate of HDAC6 [241], accelerated transport of polyU-proteins into aggregates and to autophagosomes [240], and enhanced transport of lysosomes to autophagosomes [156]. The importance of autophagosomal transport for effective clearance of aggregated proteins has been demonstrated in HD fly and mouse models, where dynein mutations caused increased aggregate formation and decreased autophagosome-lysosome fusion [31].

Recently, autophagy was genetically manipulated in a mouse model of AD by crossing *Becn1 *heterozygous knockout mice (*Becn1*^+/-^) with human amyloid precursor protein (APP) transgenic mice. *Becn1 *deficiency resulted in neurodegeneration and increased β-amyloid (Aβ) deposition in APP mice [59]. Based on these findings and new cell culture data from our lab (Jaeger *et al.*, manuscript in preparation) we propose that autophagosomes can degrade APP and thus lower Aβ accumulation [59]. On the other hand, autophagosomes contain the enzymes necessary for processing of APP into Aβ and are potential producers of this toxic peptide [58]. A decisive factor that determines whether autophagy reduces or promotes Aβ accumulation might be the speed of autophagosomal turnover and the clearance of autophagic vesicles. Both are impaired under disease conditions [62]. Disturbances in autophagy initiation due to insufficient BECN1 levels could cause expansion of the endosomal-lysosomal system, producing a high load of potentially Aβ generating vacuoles. Interestingly, two APP mouse models for AD have been analyzed for changes in Becn1 levels, but no differences were detected [59]. These findings hint at an autophagy dysfunction upstream of APP pathology in AD.

CMA is also clearly involved in chronic neurodegenerative diseases, most prominently in PD: HSP90 levels are increased in human PD brains and are correlated with the levels of insoluble α-Synuclein [242]. In the same study, immunohistochemistry and EM show that HSP90 co-localizes with α-synuclein in Lewy bodies, Lewy neurites, and glia cell inclusions, both in PD patients and α-synuclein transgenic mice. Furthermore, HSP90 and HSC70 co-immunoprecipitate with α-synuclein in cell culture [242]. While this could indicate increased (protective) CMA in PD, a recent gene expression profiling of substantia nigra tissue from sporadic PD patients revealed reduced expression of UPS proteins and reduced HSC70 [243]. At some point during disease progression, HSP90 may be sequestered into α-synuclein aggregates and deactivated, thus reducing CMA activity.

A landmark study identified α-synuclein as a target for CMA and demonstrated that the PD associated mutations A53T and A30P cause α-synuclein to bind to the CMA receptor and inhibit both the degradation of the receptor itself and that of other CMA substrates [210]. While these α-synuclein mutations are relatively rare, recent findings demonstrate that post-translational modifications of wildtype α-synuclein through dopamine can cause a similar toxic gain-of-function behavior [213]. Furthermore, inhibition of CMA by lentiviral RNAi against *LAMP2 *increases the level of endogenous α-synuclein in rat cortical neurons [102]. Additionally, a link has been suggested between the PD associated mutant ubiquitin carboxyl-terminal esterase L1 (UCH-L1) and the lysosomal receptor for chaperone-mediated autophagy. This mutant UCH-L1 interacts aberrantly with LAMP2, HSC70, and HSP90, inhibits CMA and causes an increase in α-synuclein in cell culture [212].

While the role of autophagy in neurodegenerative diseases is far from being understood, the available data indicate it plays an integral role in the cellular response to intracellular protein aggregation common to these diseases. Autophagy appears impaired in the final stages of neurodegenerative diseases, whereas alterations in vacuolar trafficking are apparent in early stages, often before other histopathological changes manifest themselves. It is therefore likely that autophagy, UPS, the endosomal-lysosomal pathway, and the escalating accumulation of toxic proteins are tightly connected. Whether mutant or misfolded proteins are causing the changes in vacuolar trafficking and later autophagy or whether abnormalities in these protein degradation pathways precede protein aggregation remains to be shown.

## Autophagy in acute CNS diseases and injuries

The second class of brain insults that present with an autophagy phenotype are acute injuries or stressors which activate competing cellular death and pro-survival pathways (Tab. 4). Examples include hypoxia/ischemia [80,94,104,244-247], brain trauma [65,81,84,87,95,106,248-250], experimental pharmacological injury models (kainate, methamphetamine, oxidative stress and others) [96,103,166,251-254], and trophic factor deprivation [255-259]. Similar to chronic neurodegenerative conditions, many observational studies find increased levels of autophagy proteins and/or numbers of autophagosomes after acute CNS injury such as hypoxia/ischemia or trauma [81,87,94,95,104,106,244,246,248,250].

As described in the previous chapter above, autophagy has beneficial functions in neurons that seem to be relevant for acute injury as well. For example, the autophagy inducing drug rapamycin reduced brain injury and protected neurons in a rat model of neonatal hypoxia/ischemia [80,249] or traumatic brain injury in mice [80,249]. Consistent with these findings, RNAi mediated knockdown of *bec-1*, *lgg-1*, and *lgg-2*, or mutation of *unc-51 *reduced survival after hypoxia in *C. elegans *[247].

However, in contrast to most studies in chronic degenerative models, acute pharmacologically induced injury or withdrawal of trophic support triggered cell death that involved autophagy and signs of apoptosis (Tab. 4). In support for a role in promoting cell death, inhibition of autophagy by 3-methyladenine (3-MA) treatment, decreased the toxic effects or delayed neuronal loss after noxious treatments [103,253,254,260]. Likewise, knockdown of *ATG5 *or *BECN1 *by RNAi reduced cell death in photoreceptor cells that were exposed to oxidative stress [253]. Maybe most convincingly, *Atg7*^flox/flox^;nestin-Cre mice lacking *Atg7 *in the neuronal lineage are almost completely protected against stroke-induced neurodegeneration [245].

Why seemingly similar studies come to these opposing conclusions is not clear at this point but differences in the models, the tools used to analyze autophagy, or the time of analysis after injury could be responsible. In support of the last point, autophagy was still increased in surviving cells at the injury site one month after traumatic brain injury [106] while cells undergoing necrotic or apoptotic death (and possibly involving autophagy in its detrimental role) would likely have disappeared. It will therefore be interesting to explore whether inhibiting autophagy early or late after a traumatic brain injury may have different outcomes. In addition, a better understanding of how exactly autophagy contributes to cell death and how it interacts with necrotic and apoptotic death programs is necessary.

## Autophagy and Apoptosis

**Figure 4:**
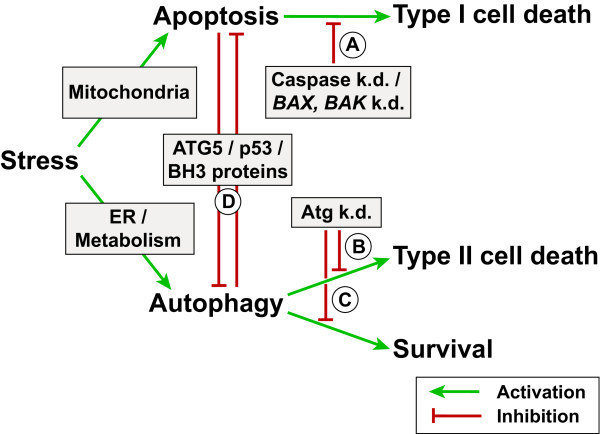
**Interaction between autophagy and apoptosis**. Cellular stressors can lead to mitochondria outer membrane permeabilization (MOMP) and subsequent cytochrome c release and apoptosis, while nutrient deficiency or ER stress can cause autophagy activation. Under physiological conditions autophagy and apoptosis keep each other inactive through mutual inhibition. A strong apoptotic stimulus (for example DNA damage, death-receptor stimulation, or cytokine deprivation) can drive a cell into apoptotic 'type I' cell death. If apoptosis is inhibited under such conditions (by caspase knockout or *Bax/Bak *knockout, [A]), autophagy can become activated and result in a delayed 'type II' cell death through degradation of most cytoplasmic cell components and organelles. Under these circumstances the knockdown of autophagy related genes [B] reduces cell death. Autophagy can become activated through ER stress (for example accumulation of misfolded proteins in the ER, intracellular calcium release from the ER) or nutrient deficiency. The cell then ensures survival by enhancing metabolic recycling through autophagy and adapting to the new nutrient conditions. Knockdown of autophagy genes in such a situation leads to an increase in apoptotic 'type I' cell death [C]. The crosstalk between autophagy and apoptosis [D] is mediated via proteolytic processing of ATG5, the transcription factor p53, and the binding and subcellular localization of BCL2 family proteins with BH3 domains. For further details, please refer to the references in the text.

As described in the previous chapters, autophagy in the CNS can be protective under some circumstances, while it leads to cell death in others. Furthermore the resulting cell death can be either apoptotic (type I cell death) or autophagic (type II cell death), depending on the cellular setting and inducing stressor (see also reviews [133,158]). This dichotomous role of autophagy is the result of a complex relationship between the autophagy and apoptosis pathways (Fig. 4). While some mixed phenotypes have been reported [261-263], autophagy and apoptosis ultimately develop in a mutually exclusive way and appear to inhibit each other [264-267].

Strong evidence for a role of autophagy as an alternative cell death mechanism comes from mice deficient in apoptosis. One of the key features of apoptotic cell death is the mitochondrial outer membrane permeabilization (MOMP), which requires the two BCL2 family proteins BAX and BAK1. Cells from *Bax*^-/-^*Bak*^-/- ^knockout mice are resistant to various apoptotic stimuli, but can die through a delayed autophagic cell death in response to DNA damage [268]. Autophagic cell death can also be observed after caspase inhibition, a treatment that disrupts normal apoptosis [266]. Conversely, inhibition of autophagy via RNAi targeting various autophagy genes (*ATG5*, *ATG7*, *BECN1*) can reduce autophagic cell death in certain situations [268-270].

In contrast to its function as a cell death mechanism, autophagy is induced under starvation conditions to supply the cell's metabolic needs. Under these conditions, inhibition of autophagy results in cell death [8]. Even without starvation, loss of autophagy itself (as in the *Atg5*^-/- ^or *Atg7*^-/- ^knockout mice) is sufficient to cause neuronal apoptosis [60,61], and it has been suggested that autophagy is primarily a pro-survival pathway [271].

It has been shown that autophagy and apoptosis share common inducers such as reactive oxidative species (ROS), ceramide, and intracellular calcium [188,272-275]. The two pathways are further linked through ATG5 proteolysis [275], the transcription factor p53 [276], and the BCL2 protein family (via BECN1) [277] (Fig. 4). How the balance between autophagy and apoptosis is maintained in neurons requires further investigation.

## Concluding remarks

Unknown to most neuroscientists just a few years ago, autophagy has gained increasing attention not only from translational researchers but also from basic neuroscientists interested in neuronal cell biology. Consequently, there are few answers as to the role and relevance of autophagy in neurons, let alone in glia cells, and very few genetic *in vivo *studies have been conducted to investigate its role in neurological disease. Nevertheless, it seems clear that neurons require autophagy for normal function and that neuronal stress will rapidly trigger this pathway (see Appendix 1: Key Observations). There is growing consent that intraneuronal protein aggregates trigger autophagy and that this response is beneficial – at least in its intent. This notion is supported by a limited number of pharmacological and genetic studies in animal models, which demonstrate that reduced autophagy promotes neurodegenerative disease while increased autophagy is beneficial. In contrast, work from stroke models and other acute forms of neural injury indicate that autophagy can be detrimental in such circumstances and promotes cell death. It will be necessary to employ state of the art genetic and molecular tools to dissect the role of autophagy in normal and pathological conditions in cell culture and in mammalian disease models (see Appendix 2: Critical Next Steps). Conditional knockout mice are being developed or are already available to target autophagy not only in neurons but also in astrocytes, oligodendrocytes and microglia. Such studies are likely to add additional complexity to our understanding of autophagy but they may also uncover new therapeutic opportunities. Self-eating, after all, does not equate with self-destruction but may in fact be a powerful survival pathway for the cell, and as such, of key importance to neurodegeneration or neuroprotection.

## Abbreviations

3-MA: 3-Methyladenine; Atg: Autophagy related genes; AD/PD/HD: Alzheimer/Parkinson/Huntington disease; APP: Amyloid precursor protein; AV: Autophagic vesicles; CMA: Chaperone-mediated autophagy; CNS: Central nervous system; EM: Electron microscopy; ER: Endoplasmatic reticulum; htt: Huntingtin; MOMP: Mitochondrial outer membrane permeabilization: MVB: Multivesicular body; NGF: Nerve growth factor; PE: Phosphoethanolamine; PI3K: Phosphoinositide 3-kinase; polyQ/polyA/polyU: Proteins with long sequences of Glu/Ala or that are ubiquitin decorated; Rap: Rapamycin: ROS: Reactive oxidative species: UPS: Ubiquitin-proteasome system; WM: Wortmannin;

## Competing interests

The authors declare that they have no competing interests.

## Appendix 1

### Key Observations

• Autophagy plays a crucial role in maintaining neuronal homeostasis through clearance of defective organelles and unfolded/aggregating proteins. Knockout of autophagy pathway genes leads to accumulation of poly-ubiquitinated protein aggregates and can result in neurodegeneration, and motor and behavioral deficits in mice.

• Autophagy interacts with other protein degradation and vesicular trafficking pathways. While autophagy can at least partially substitute for reduced proteasomal activity and vice versa, the disturbance of the endosomal-lysosomal system disrupts autophagy and reduced autophagy impairs endosomal-lysosomal trafficking.

• Autophagy clears neurotoxic proteins. Activation of autophagy reduces the toxicity of aggregation prone proteins, while inhibition of autophagy impairs their clearance and causes enhanced cellular stress and neurodegeneration.

• Autophagy can be a cellular death pathway, which is activated in neurons after acute injury and inhibition of autophagy under those conditions can reduce neurodegeneration.

• Autophagy is impaired in the final stages of most neurodegenerative diseases.

## Appendix 2

### Critical Next Steps

• What is the sequence of events? Impaired autophagy is a histopathological hallmark of many neurodegenerative diseases. But it is unknown if autophagy is first impaired, contributing to the disease early on, or if autophagy is highly active to fight the disease and is overwhelmed in the end. The use of inducible knockout animals crossed with traditional disease models or RNAi against autophagy genes in different disease stages could help to elucidate this problem.

• Which autophagy genes are involved? Autophagy is mediated through an evolutionary conserved pathway involving more than 20 proteins. Several of them link autophagy to other important cellular pathways such as apoptosis, the ubiquitin/proteasome system, the endosomal-lysosomal system, and vesicle and receptor trafficking. Which proteins are involved in neurodegeneration is not well understood. Careful analysis of autophagy activity, and mRNA and protein levels of central autophagy genes in tissue from human patients and animal models could help us identify the key players.

• What genetic mutations are associated with autophagy and altered susceptibility to neurodegeneration? While some data exist about mutations in disease-associated genes that interact with autophagy, no mutations in human autophagy genes that cause neurodegeneration are known so far. If autophagy plays a central role in protein clearance, the identification of change-of-function mutations in autophagy genes would be essential to define "autophagosomal diseases".

• How can autophagy be modulated to enhance clinical outcome? The discovery of drugs beyond rapamycin to enhance autophagy has made substantial progress. Because autophagy is linked with multiple intracellular pathways, the identification and functional characterization of key proteins that specifically control only limited aspects of this interplay could help design more precise modulators of autophagic activity, with lessened effects on connected pathways.

## Authors' contributions

PAJ collected references, generated the artwork, and wrote the manuscript with input from TWC.

## Note

Throughout this review approved human gene and protein names are used to describe experiments and general observations (independent of the actual source species of the cells or the findings discussed). Only for targeted disruption of endogenous genes (such as knockout mice) species-specific nomenclature is used.
